# Special Issue: Structure, Properties and Applications of Polymeric Foams

**DOI:** 10.3390/ma14061474

**Published:** 2021-03-17

**Authors:** Aleksander Hejna

**Affiliations:** Department of Polymer Technology, Faculty of Chemistry, Gdańsk University of Technology, Gabriela Narutowicza 11/12, 80-233 Gdańsk, Poland; aleksander.hejna@pg.edu.pl

The Special Issue “Structure, Properties and Applications of Polymeric Foams” aimed to gather the numerous reports associated with the different aspects of polymeric foams. First of all, The Guest Editor would like to express gratitude to the Editors-in-Chief of *Materials* for the opportunity to create and run the Special Issue, as well as to all the authors from the whole world who contributed with their high quality papers, and reviewers, who helped to enhance the level of manuscripts. Finally, the Section Managing Editor, Ms. Yulia Zhao, should be acknowledged for the excellent management of the editorial process.

Focusing on the topic of the Special Issue, foaming of polymeric materials enables weight reduction, very important from the economic point of view, but most of all it should be considered as an excellent means to provide new properties to various polymeric materials [1,2]. Permanent developments in foaming technologies allow manufacturing of foams with micro- and even nano-sized pores, expanding the already very wide range of their applications. Moreover, recent advances allow the preparation of functionally graded foams, which are characterized by the engineered porosity distribution, imitating the cellular materials created by nature, e.g., plant stems or bones. Recent progress in processing of such materials was comprehensively summarized by Suethao et al. [3]. Nevertheless, it is important to remember about the most conventional applications of polymeric foams as insulation materials, which still require substantial research, e.g. considering the heat transfer mechanism, investigated by Blazejczyk et al. [4].

Moreover, having in mind the ongoing trends and law regulations, it is important to remember the environmental impact of polymeric foams. Recent technological developments often involve biodegradability of foams, application of environmentally friendly raw materials, and innovative recycling methods [5]. Such an approach was strongly emphasized by Abolins et al. [6], who described the innovative transformation of tall oil fatty acids into bio-polyols. Their application in manufacturing of rigid polyurethane foams could enable substitution of petroleum-based materials and reduction in foams’ environmental impact without the deterioration of thermal insulation performance. Another interesting paper dealing with the biopolyols from renewable resources was published by Kosmela et al. [7]. They used biopolyol (obtained by crude glycerol liquefaction of *Enteromorpha* macroalgae and *Zostera marina* seagrass originated from Baltic Sea) to prepare rigid polyurethane foams. Except for the conclusions related to the benefits of the environmentally-friendly approach, it is worth mentioning the excellent application of micro-computed tomography for the evaluation of foams’ cellular structure. When combined with the finite element analysis, it can be used to investigate the relationship between the macroscopic mechanical properties and microscopic foam structure, as presented in the excellent paper of Iizuka et al. [8]. Deeper analysis of this relationship can enable the adjustment of the manufacturing process to obtain the desired microstructure of foams, which directly affects their performance. As a result, tuning the microstructure of foams could be used to acquire desired global materials properties [9].

Alongside for the application of renewable raw materials, the use of recycled ones should be emphasized considering the current pro-ecological trends, as in the work of Hejna et al. [10] dealing with structure and performance of flexible foamed polyurethane/ground tire rubber composites.

Summarizing, the Guest Editor believes that the collection of the articles presented as a Special Issue “Structure, Properties and Applications of Polymeric Foams” perfectly shows the multitude of foams’ applications and potential research directions associated with these materials. 
